# Comprehensive *in vitro* and *in vivo* studies of novel melt-derived Nb-substituted 45S5 bioglass reveal its enhanced bioactive properties for bone healing

**DOI:** 10.1038/s41598-018-31114-0

**Published:** 2018-08-24

**Authors:** Lucas Souza, João Henrique Lopes, Davi Encarnação, Italo Odone Mazali, Richard Alan Martin, José Angelo Camilli, Celso Aparecido Bertran

**Affiliations:** 1 Department of Structural and Functional Biology, Institute of Biology, University of Campinas – UNICAMP, 13083-862 Campinas, SP Brazil; 2 Department of Physical Chemistry, Institute of Chemistry, University of Campinas – UNICAMP, P.O. Box 6154, 13083-970 Campinas, SP Brazil; 3 Laboratory of Functional Materials, Department of Inorganic Chemistry, Institute of Chemistry, University of Campinas – UNICAMP, P.O. Box 6154, 13083-970 Campinas, SP Brazil; 4 School of Engineering & Aston Research Centre for Healthy Ageing, Aston University, B47ET Birmingham, United Kingdom

## Abstract

The present work presents and discusses the results of a comprehensive study on the bioactive properties of Nb-substituted silicate glass derived from 45S5 bioglass. *In vitro* and *in vivo* experiments were performed. We undertook three different types of *in vitro* analyses: (i) investigation of the kinetics of chemical reactivity and the bioactivity of Nb-substituted glass in simulated body fluid (SBF) by ^31^P MASNMR spectroscopy, (ii) determination of ionic leaching profiles in buffered solution by inductively coupled plasma optical emission spectrometry (ICP-OES), and (iii) assessment of the compatibility and osteogenic differentiation of human embryonic stem cells (hESCs) treated with dissolution products of different compositions of Nb-substituted glass. The results revealed that Nb-substituted glass is not toxic to hESCs. Moreover, adding up to 1.3 mol% of Nb_2_O_5_ to 45S5 bioglass significantly enhanced its osteogenic capacity. For the *in vivo* experiments, trial glass rods were implanted into circular defects in rat tibia in order to evaluate their biocompatibility and bioactivity. Results showed all Nb-containing glass was biocompatible and that the addition of 1.3 mol% of Nb_2_O_5_, replacing phosphorous, increases the osteostimulation of bioglass. Therefore, these results support the assertion that Nb-substituted glass is suitable for biomedical applications.

## Introduction

Fractures, bone tumours, periodontal diseases, and degenerative cartilage disorders disrupt daily activities of people. Therapeutic approaches to these pathologic conditions prompted the development of materials that could replace bone and joint tissues^1–3^. Many materials were produced based on metals, such as aluminium, zirconia and titanium, and were used in orthopaedics for manufacturing prosthesis, plates, nails, screws, and other such components. However, as these materials in general have a lifespan of around 10–20 years and human life expectancy has increased, these implants frequently deteriorate prior the patient’s death^4–6^.

At the beginning of the 1970s, the researcher Larry. L. Hench and his colleagues developed the first bioactive material, the 45S5 bioglass (Bioglass^®^). This biomaterial not only induced no inflammatory response in the body, but also formed strong bonds with bone and soft tissues, optimizing stability of the implant and extending its durability^6–8^. This material is a bioactive glass belonging to the system SiO_2_-CaO-Na_2_O-P_2_O_5_ (46.1 mol% SiO_2_, 26.9 mol% CaO, 24.4 mol% Na_2_O, and 2.6 mol% P_2_O_5_)^9^. The strong interface between biomaterial and tissue is established through chemical reactions that occur on the surface of the bioglass, concomitant with biological events leading to the formation of a nanocrystalline calcium phosphate hybrid composite permeated by biological molecules^7,8^.

Several articles have been published, reviewing the literature discussing the effect of 45S5 bioglass composition on bioactivity, angiogenesis, osteostimulation, osteoconduction, and other important properties for bone regeneration^10–14^. As the chemical composition of glass dictates its effects on living tissues, many other variations of the original Bioglass^®^ have been manufactured and investigated for use as biomaterial; some of them showed high bioactivity, for example 58 S, S53P4, and 70S30C^2,15–19^.

Furthermore, several types of transition metal-doped soda lime silica glass have been prepared, and their bioactivity has been evaluated for biomedical applications^20,21^. Among the various vitreous compositions investigated, glass containing niobium oxide have received special attention, since the presence of niobium species has been associated with improvements in bioactive and mechanical properties^22–26^. Moreover, recent works have reported the ability of niobium ions to enhance differentiation and mineralization of osteogenic cells *in vitro*^24,25^. Metallic niobium has been used in titanium alloys for endosseous implants, aiming to increase the biocompatibility and corrosion resistance, with superior fatigue resistance^27^. Similar behaviour of phosphate bioactive glass was observed on adding Nb_2_O_5_^23,26^. It is noteworthy, however, that the Nb_2_O_5_ is an effective nucleating agent and generally leads to the development of glass-ceramic in silica-poor compositions (inverted glass), such as 45S5^28^.

In our previous study, the structures of two series of bioglass (derived from 45S5 Bioglass^®^ composition) with varying niobium oxide contents were exhaustively investigated by multinuclear ^29^Si, ^31^P, and ^23^Na solid-state MAS NMR and Raman spectroscopy, and some physical properties of the glass were also studied^29^. The structural investigation of bioglass resulting from the substitution of P_2_O_5_ by Nb_2_O_5_ revealed that Nb_2_O_5_ participates in the silicate network by breaking the Si-O-Si bonds to form structures, such as -Si-O-Nb-O-Si- chains. These alterations significantly changed the glass dissolution rate and magnitude, which is of interest for understanding its higher bioactivity^29,30^.

Even though Nb-substituted silicate glass appears to possess interesting bioactive properties, its effect over pluripotent cells and bone regeneration has not been investigated till date. In view of this, the objective of this study was to evaluate the *in vitro* and *in vivo* effects of different compositions of Nb-substituted bioglass on properties, namely, cytotoxicity, osteoinduction, osteoconduction, and osteostimulation, that are essential for osteointegration of medical materials for bone replacement. The *in vitro* approach consisted of treating human embryonic stem cells (hESCs) with the dissolution products from the glass and verifying cell viability and osteogenic differentiation. For the *in vivo* assay, glass rods were implanted into circular defects in rat tibia and bone formation was quantified. In addition, the leaching profile of Si, Ca, Na, P, and Nb species from the bioglass, and the kinetics of structural transformations of different compositions of Nb-substituted silicate glass were investigated.

## Results and Discussion

### Dissolution studies

The leaching rate of Na, Ca, Si, and P ions from bioglass in the earliest stages of the bioreactivity is of utmost importance since the results of the leaching processes will control the subsequent biomineralization. To examine leaching rates, two promising compositions of Nb_2_O_5_-containing bioactive glass were studied. The 45S5 bioglass composition is also presented for the purpose of comparison. The nomenclature and chemical composition of all samples studied in this work are shown in Table 1.

**Table 1:** Glass compositions of BG45S5 and Nb-substituted 45S5 bioglass.

Glass					
	SiO_2_	CaO	Na_2_O	P_2_O_5_	Nb_2_O_5_
BG45S5	*46,1*	*26,9*	*24,4*	*2,6*	*—*
BGPN2.6	*46,1*	*26,9*	*24,4*	*—*	*2,6*
BGPN1.3	*46,1*	*26,9*	*24,4*	*1,3*	*1,3*

**Figure 1:**
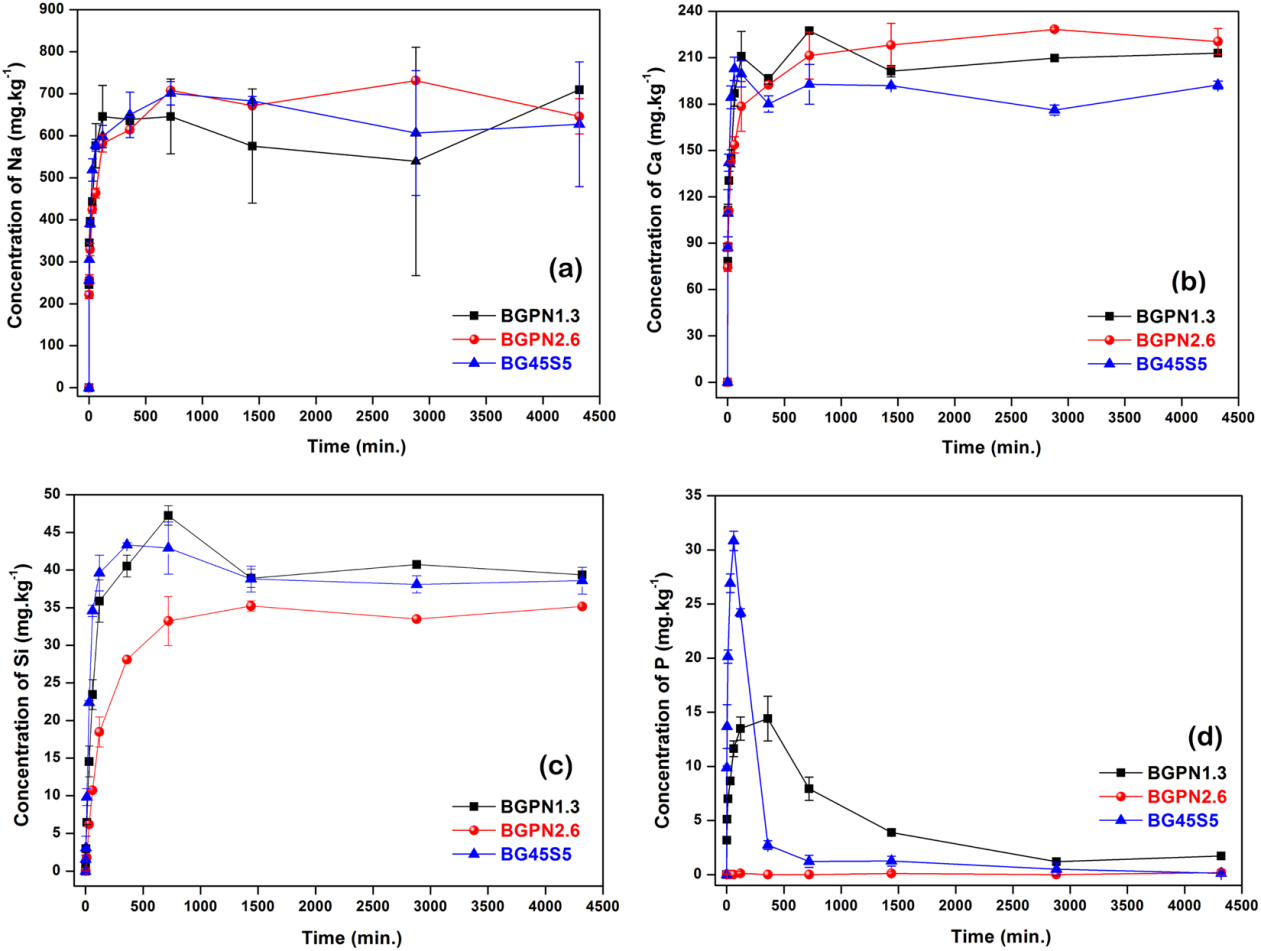
ICP data: ion release vs. time in 50.69 mM HEPES solution at pH 7.40 for BG45S5 and Nb-substituted bioactive glass. The data displayed in **(a–d)** are related to leached Na, Ca, Si, and P species from glass derived from substitution of P_2_O_5_ for Nb_2_O_5_.

**Figure 2:**
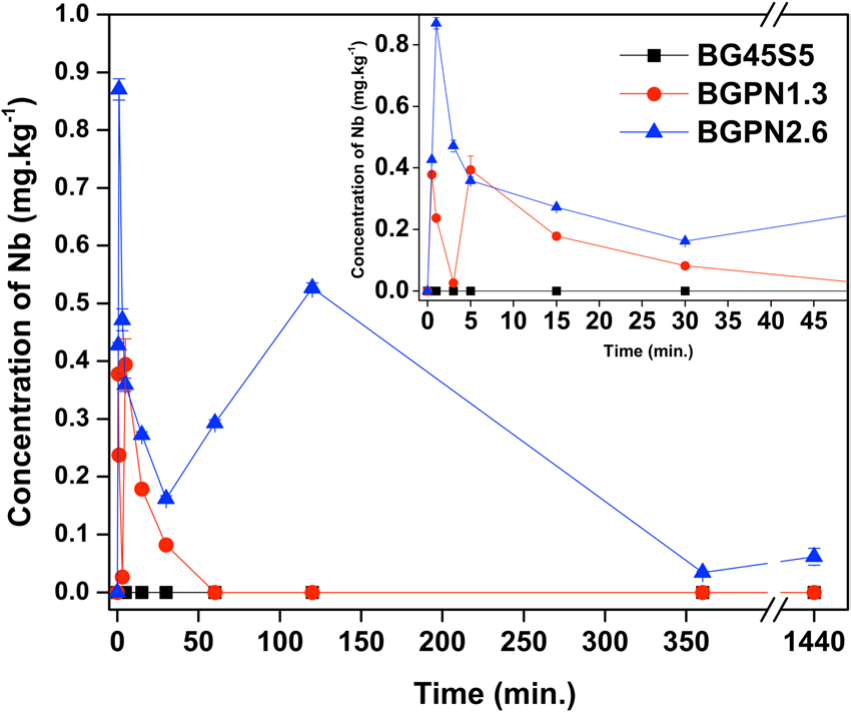
ICP data: Nb ion release vs. time in 50.69 mM HEPES solution at pH 7.40 for BG45S5 and Nb-substituted bioactive glass. The amounts of niobium determined by ICP-OES is consistent with the niobium content in each sample, i.e. BGPN2.6 exhibits a higher peak of niobium concentration compared to the BGPN1.3 bioglass. As expected, no niobium was detected for BG45S5.

The effect of Nb_2_O_5_-content on the glass solubility was studied by time-dependence of species release from bioglass particles dispersed in 50.69 mM HEPES buffered solution (pH = 7.4). The leaching curves for Na, Ca, Si, P (Fig. 1) and Nb (Fig. 2) species were determined by using inductively coupled plasma optical emission spectrometry (ICP-OES).

We found that the initial elemental concentrations of sodium, calcium, silicon, and phosphorus, up to about 120 min for all compositions, are a linear function of the square root of time, which indicates a diffusion-controlled process (Supplementary Information – Fig. SI1a–d)^8^. The shape of the curves is consistent with Hench’s mechanism, which describes all events on bioglass surface in contact with body fluid in five steps: (i) rapid exchange of Na^+^/Ca^2+^ from glass network with H_3_O^+^ from solution, (ii) loss of soluble silica/ formation of silanol groups at the glass solution interface, (iii) condensation and repolymerization of a SiO_2_-rich layer, (iv) growth of the amorphous CaO-P_2_O_5_-rich film by incorporation of soluble calcium and phosphates from solution, (v) crystallization of the amorphous CaO-P_2_O_5_ film by incorporation of OH^−^ and CO_3_^2−^ anions from solution^4,7,31^.

The leaching profiles of network modifier ions, Na^+^ and Ca^+^, for BG45S5 and Nb_2_O_5_-substituted bioglass showed an abrupt increase, reaching a maximum concentration value for these species at around 2 hours and remaining constant for longer times (Fig. 1a,b**)**. The release curves showed that the amount and rate at which Na and Ca ions are leached from the glass networks were similar to each other, in particular, within the first hours after dipping. This result is of extreme importance because it shows that the replacement of P_2_O_5_ by the Nb_2_O_5_ in the 45S5 composition did not alter the onset of the cascade of events, which includes ion exchange between Na^+^ and Ca^2+^ ions and H_3_O^+^, as described by the Hench mechanism for biomineralization^7,31,32^. In addition, recognizing that sodium niobate and/or calcium niobate are highly water insoluble species close to the neutral pH, these results corroborate with those previously reported on structural data for these glassy compositions^29^, suggesting that niobium (NbO_6_) would be behaving as a network former by establishing chemical bonds with silicon tetrahedral (Si-O-Nb-O-Si) in the glass backbone (chain of alternating Si and O atoms).

Conversely, the leaching curves for the species of silicon and phosphorus showed peculiar behaviours for each bioglass composition (Fig. 1c,d). The amounts of soluble silicon species determined after the first hour of immersion were similar, except for the BGPN2.6 sample, which exhibited a reduced concentration of silicon. This result indicates the presence of Si-O-Nb bonds, where the NbO_6_ octahedra replaces part of the SiO_2_ tetrahedra in the glass network. Evidently, an increase in niobium content in the composition must result in a greater number of Nb-O-Si bonds, so that such structures may act as traps for the silicon species in the glass matrix, preventing and delaying their leaching. The release profile of phosphorus suggests a maximum concentration (approximately the solubility limit), from which there is a dramatic reduction, possibly associated with the removal of some of the soluble phosphate species to form the calcium phosphate layer on the bioglass surface (Fig. 1d). Although the rate at which species of phosphorus are released into solution is similar to BG45S5 and BGPN1.3 samples, the removal of phosphorus from solution is slower for the BGPN1.3, which can be attributed to its lower P_2_O_5_ concentration (phosphorus concentration was not high enough to reach the limit solubility of calcium phosphate). As expected, no appreciable amount of phosphate species was observed for BGPN2.6.

The leaching curves for the species of niobium exhibit similar profiles to those observed for phosphorus species (Fig. 2).

At early soaking times, it is possible to observe an abrupt increase in the niobium concentration in the medium, reaching a maximum value, followed by a dramatic reduction of concentration value. This initial burst can be credited for the leaching of niobium species near surface and/or release of isolated NbO_6_ units isolated niobium octahedral in the vitreous matrix, which are more labile since their release does not require extra energy to break bonds. The maximum niobium species concentration determined for samples BGPN1.3 and BGPN2.6 was 0.4 ppm after 30 s and 0.9 ppm after 1 minute, respectively. The drastic decrease in niobium content observed for samples BGPN1.3 and BGPN2.6 is closely related to the very low solubility of niobium species at pH 7.4 in aqueous medium. The presence of a second peak of Nb concentration was observed for the BGPN1.3 and BGPN2.6 bioglass of 0.4 ppm after 5 minutes and 0.5 ppm after 120 minutes, respectively. This second event is directly related to the leaching of isolated niobium species in the bulk and/or Nb species that were more firmly trapped in the vitreous network. Again, the abrupt decrease in niobium concentration is due to the fact that the product solubility is small for niobium species. In fact, the concentration of niobium after each release peak tends to go to zero, confirming that the residence time of the Nb species in the solution body is short. Consequently, almost all niobium, if not all, may be retained in aggregates in the solution (removed during the filtration step) or in silica gel formed at the bioglass/solution interface.

Generally, it is common to use chelating agents such as citrates and oxalates to increase the solubility of niobium species^33,34^. It is noteworthy that all the studies of leaching presented in this report were carried out using a medium containing HEPES, which is an often-used agent in studies, among several other reasons because it does not chelate with metals. Obata *et al*.^25^ investigated a glass series belonging to 60CaO-30P_2_O_5_-(10−x)Na_2_O-xNb_2_O_5_ - system (mol%, x = 0–10) and quantified the content of niobium in the leachate by ICP-OES after a few hours of immersion. However, in this study, the dissolution of the glass was carried out in a complex culture medium (minimal medium culture + bovine serum) containing various chelating ligands, which may explain the presence of niobium in the solution. It is also worth noting that these phosphate glasses studied by Obata and co-workers can dissolve completely in aqueous solution giving ionic species, which may form precipitates upon reaching the maximum solubility (solubility product constant - K_sp_). Moreover, another important point deserving attention in the work of Obata is that the culture medium has not apparently been filtered, consequently, the undissolved vitreous particles and the possibly formed ionic-molecular agglomerates, both containing niobium, remain in the medium. Hence, not only the niobium species chelated in the medium, but also those present in the glass particles or aggregates are released into the solution during the oxidation step of the organic material (commonly under acidic conditions) required for ICP-OES analysis were quantified^25^. Thus, the low solubility of the species of niobium in solution together with the filtration step using membranes of 0.22 μm, and the absence of chelating agents in the present study suggests that the Si-O-Nb bond undergoes hydrolysis, but only small amount of niobium goes into the solution, while the leached niobium tends to form complex structures (niobium-silica gel layer), which is retained on the glass surface.

### Apatite layer formation ability by ^31^P MAS NMR spectroscopy

**Figure 3:**
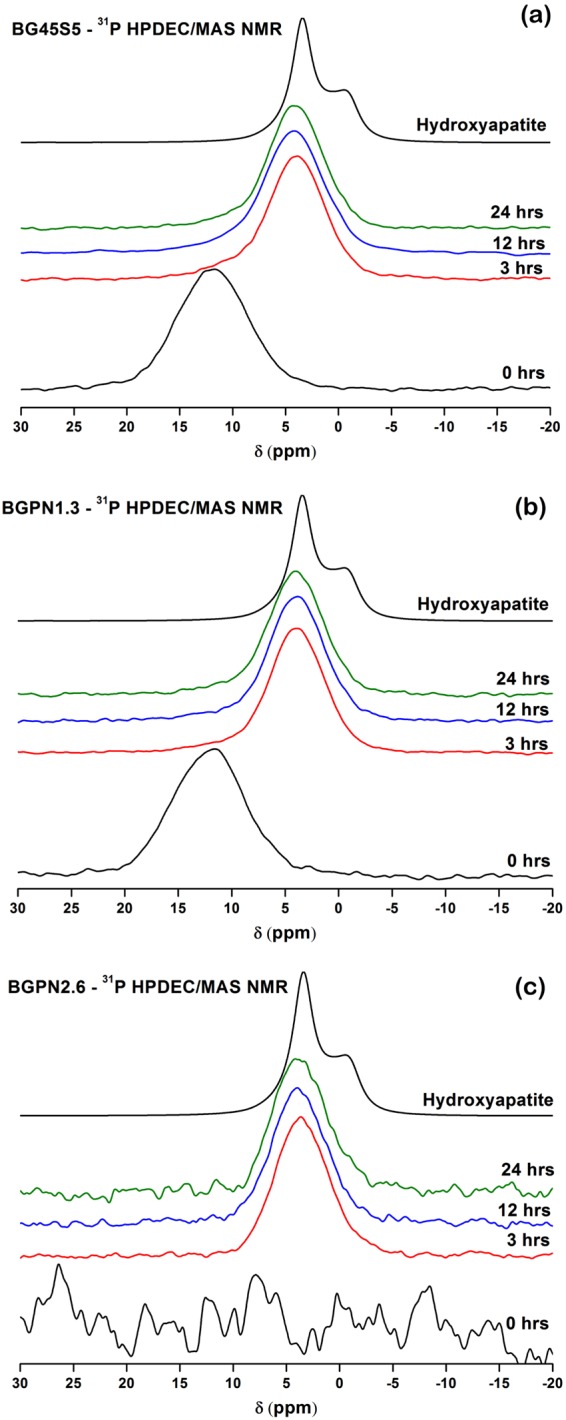
^31^P MAS-NMR spectra for the different glass compositions, recorded at a magnetic field strength of 9.4 T and a spinning speed of 10 kHz: **(a)** BG45S5, **(b)** BGPN1.3, and **(c)** BGPN2.6. The chemical environmental for ^31^P shifts progressively evolves to lower values of $\delta$, reaching a peak position similar to the ^31^P spectrum observed in the hydroxyapatite control. The absence of phosphorus in BGPN2.6 did not prevent the calcium phosphate layer formation on its surface.

The development of an apatite layer on the bioglass surface in the physiological environment (even under simulated conditions) is a common feature among all the bioactive vitreous compositions described in the literature^7,8,16,35^. Therefore, the time required for this layer to be established has been an initial parameter for assessing and ranking the different vitreous compositions in the bioactivity domain, since apatite is a cell-friendly environment^8,18^. In contrast to other analysis methods, MAS NMR spectroscopy is a powerful, univocal and sensitive tool for studying changes in the chemical environment of phosphorus, capable of identifying subtle changes in local structural arrangement of phosphorus even for early soaking times. The partial dissolution and apatite formation on the bioglass surface in simulated body fluid (SBF) has been verified by ^31^P MAS NMR (Fig. 3(a–d)**)**.

The ^31^P MAS NMR spectrum of BG45S5 consists of one broad resonance with linewidth of 7.9 ppm and peak maximum at 9.0 ppm assigned to isolated orthophosphate units, which coordinate modifier cations (Na^+^ and Ca^2+^), subtracting them from the silicate network^29,30^. The ^31^P resonance spectra for the glass compositions containing Nb_2_O_5_ illustrate a similar spectrum to BG45S5 with one broad resonance and asymmetric peak with a chemical shift ranging from 18 to −3 ppm with an approximate linewidth of 8 ppm.

^31^P MAS NMR peak positions and full width half maximums (FWHM) for BG45S5 and Nb-substituted glass, unreacted and after 3, 12, and 24 hours of immersion in SBF are listed Table 2. ^31^P MAS NMR spectrum of commercial hydroxyapatite was added as a reference for the phase of calcium phosphate formed.

**Table 2:** 31P MAS NMR peak positions and full width half maxima (FWHM) for BG45S5, Nb-substituted 45S5 bioglass and commercial hydroxyapatite.

Glass	^31^P MAS NMR							
	Immersion time in SBF (hours)							
	0		3		12		24	
	Peak (ppm)	FWHM (ppm)	Peak (ppm)	FWHM (ppm)	Peak (ppm)	FWHM (ppm)	Peak (ppm)	FWHM (ppm)
BG45S5	*11.9*	*7.9*	*4.0*	*6.2*	*4.3*	*6.6*	*4.0*	*6.1*
BGPN1.3	*11.6*	*7.9*	*3.9*	*6.0*	*3.9*	*5.9*	*4.0*	*6.0*
BGPN2.6	*—*	*—*	*3.5*	*5.9*	*3.8*	*5.9*	*3.7*	*5.8*
*Reference*								
Hydroxyapatite	*3.4*	*2.7*	*—*	*—*	*—*	*—*	*—*	*—*

In general, ^31^P MAS NMR spectra for all glass exhibit the same trends, which can be summarised by the chemical shift to lower frequencies and a decrease in FWHM values. Such spectral changes are intimately related to partial degradation of glass and precipitation of amorphous calcium phosphate and its progressive crystallization^30^.

^31^P MAS NMR spectra for BG45S5 **(**Fig. 3a**)** and BGPN1.3 **(**Fig. 3b**)** after 3 hours of incubation in SBF showed a main peak at ~4 ppm with slight asymmetry on the left-hand side of the peak owing to the residual glass fraction. This subtle spectral feature was not observed for BGPN2.6 **(**Fig. 3c**)**, which exhibited a single and symmetrical peak at 3.6 ppm, confirming that the presence of P_2_O_5_ in the glass bulk is not mandatory for precipitation of calcium phosphate layer. Although subtle, the changes observed in the spectra in the range of 3–24 hours are related mainly to the gradual increase in the crystallinity of calcium phosphate (increase of long-range order). The ^31^P MAS NMR spectra after 24 hours in SBF for all the glass compositions show chemical shifts and bandwidths similar to those observed for commercial hydroxyapatite (top spectrum in Fig. 3c). These results indicate that glass containing niobium leads to the formation of a calcium phosphate with a higher degree of crystallinity compared to BG45S5, with similar soaking time in SBF. In fact, it has been reported that Nb-OH groups in the amorphous phase of sol-gel-derived niobium oxide gels are very effective for apatite nucleation, which is a friendly environment for cells^36^.

### Biocompatibility assessment

**Figure 4:**
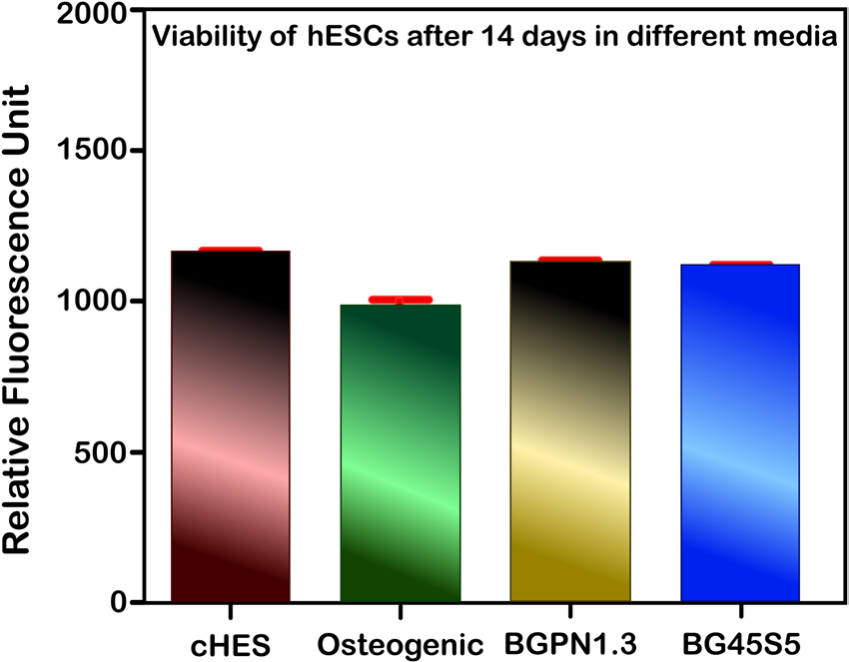
Relative fluorescence of hESCs stained with 5 μM of resazurin after 14 days of treatment with different culture media. There was no significant difference between the groups, which shows that none of the media were cytotoxic for this cell line. This study was carried out only for the BG45S5 and BGPN1.3 samples. cHES: conditioned human embryonic stem cells medium.

The first step when developing a new biomaterial to be used for tissue replacement is to ensure that it cannot represent a risk when implanted into the body. Therefore, we carried out *in vitro* and *in vivo* experiments to test the biocompatibility of Nb-containing glass. In the *in vitro* approach we treated human embryonic stem cells (hESCs), CCTL12, with the dissolution products of Nb-containing glass (BGPN1.3 and BGPN2.6) and the control glass (BG45S5) for 14 days and performed a resazurin assay. This assay revealed that none of the glass compositions were cytotoxic (Fig. 4).

Both, BG45S5 and BGPN1.3 glass showed similar fluorescence relative to that of the control group (cHES medium) after 14 days of treatment (P > 0.05). This result corroborates with those from some other reports^22,25,37^ that have studied the cytocompatibility of bioactive glass containing niobium; these studies concluded that the addition of this metal does not impair the compatibility of the biomaterial. One of these reports showed that when osteoblast-like cells are in direct contact with the surface of calcium phosphate invert glasses (PIGs) containing Nb_2_O_5_ they attach and spread after 6 hours on all different concentrations of soluble niobium species^25^. The same authors performed experiments with dissolution products of niobium-doped PIGs and revealed that they are not harmful for osteoblast-like cells (MC3T3-E1), and instead they stimulated some positive biological responses^25^.

Similar results were found by another study using a different cell line and a different formulation^24^. In this study, human mesenchymal stem cells (hMSCs) were cultured on niobium-doped fluorapatite glass-ceramics, and the results showed that after 3 hours, the hMSCs attached on niobium glass as well as they did on the control surface; the report also shows the scanning electron microscopy (SEM) micrographs of cells attached to the glass surface after 2–4 days^24^.

Testing cytocompatibility is not enough to ensure the safety of implants. Inorganic material may intoxicate some high-metabolic-rate organs that participate in the metabolism and excretion of the material, for example the liver and kidneys, which may lead to morphological alterations in these organs. Because of that, we calculated the relative weights of the livers and kidneys of rats that had undergone implantation of glass rods at circular defects in their tibia, and compared the mean values with a control group of rats. We observed no difference between the groups neither after 14 nor 28 post-operative days, proving that no alterations in these organs were caused by the presence of the inorganic implants (Table 3).

**Table 3:** Relative weight of liver and kidneys of rats treated with different bioactive glasses for 14 and 28 days.

Time	Groups	Liver (g)	Kidneys (g)	Body Weight (g)
14 days	Control	11.4 ± 0.2	5.17 ± 0.07	366.0
	BG45S5	11.4 ± 0.2	5.28 ± 0.06	381.0
	BGPN1.3	11.4 ± 0.1	5.10 ± 0.09	376.0
	BGPN2.6	11.2 ± 0.1	5.22 ± 0.07	373.0
	Sham	11. 5 ± 0.2	5.29 ± 0.08	387.0
28 days	Control	10.8 ± 0.1	5.0 ± 0.1	474.0
	BG45S5	10.8 ± 0.2	5.00 ± 0.07	466.0
	BGPN1.3	10.74 ± 0.08	4.99 ± 0.05	434.0
	BGPN2.6	10.9 ± 0.1	5.05 ± 0.07	421.0
	Sham	10.6 ± 0.2	5.02 ± 0.04	443.0

Average values of 6 animals. represented by mean ± SEM. Considering p < 0.05 no significant difference was detected. One-way ANOVA followed by Student Newman-Keuls test. Sham = no bone defect was created.

Taken together, these results suggest that regardless of the biological model and material formulation used, Nb-substituted bioglass is biocompatible, and such bioglass have proved to be excellent candidates for biomedical applications.

### Osteogenic potential of Nb-containing bioglass

Several studies have shown that by transplanting adequate numbers of competent osteoprogenitor cells on biomaterial scaffolds, cell-based bone tissue engineering strategies may promote functional reconstruction of skeletal defects^38–40^. We chose to work with human embryonic stem cells (hESCs) because we believe these cells show some important advantages compared to other more commonly used multipotent cell types like mesenchymal stem cells (MSCs). We understand that MSCs may represent a reliable source of multipotent stem cells for bone cell transplantation. However, the fact that the numbers of adult MSCs are limited, and decline with age^41^, and because they are present at different stages of differentiation at the time of harvest^42^, their use is not without problems. Human ESCs, on the other hand, possess unlimited growth and differentiation potential, accessibility, and do not require a donor site^43^, facilitating the acquisition of a large amount of cells to be used with biomaterial scaffolds for bone regeneration.

**Figure 5:**
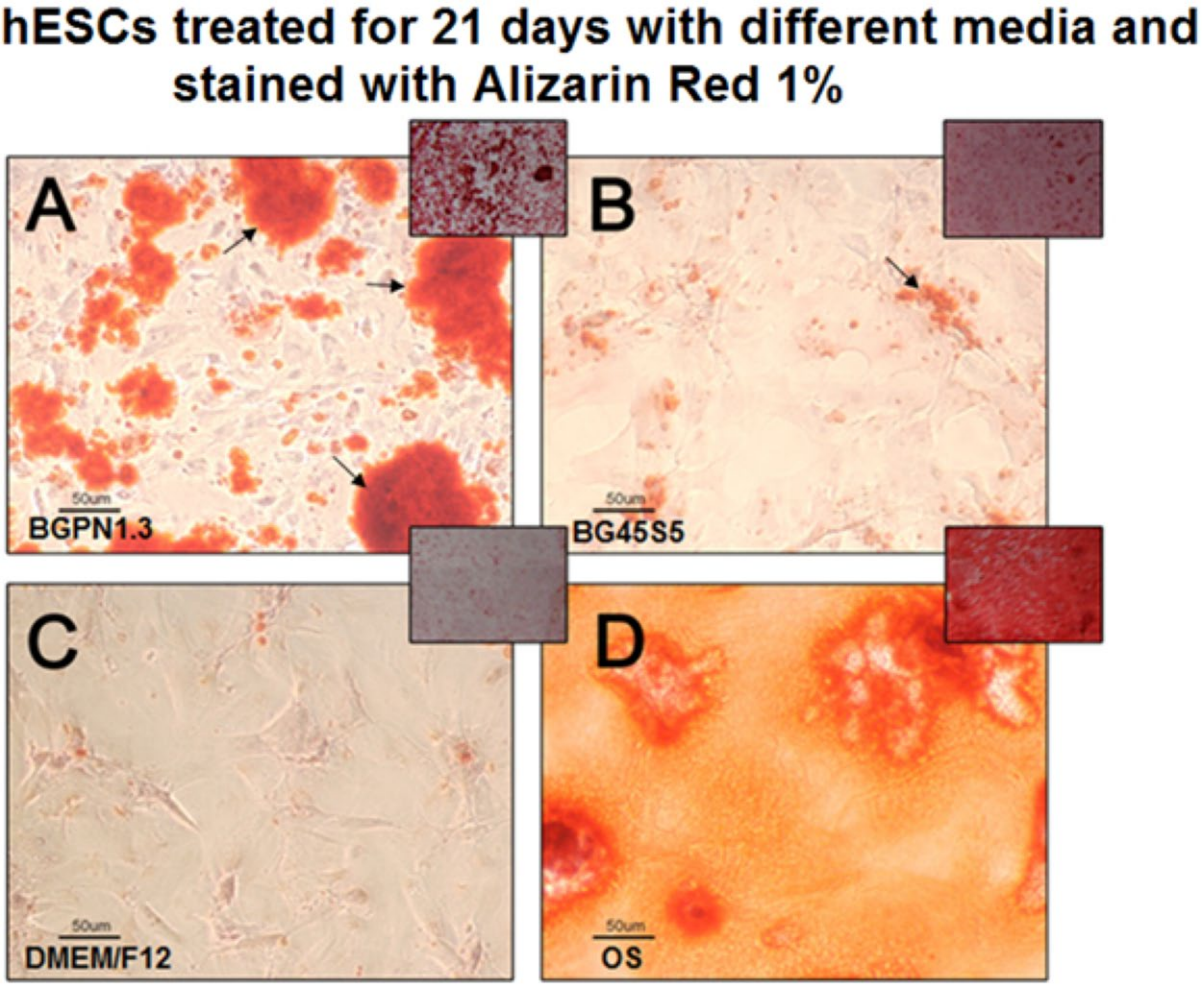
Micro and macrographs of calcium deposits within and around hESCs stained with alizarin red (1%)after 21 days of treatment with different media. The four large squares show micrographs (100x magnification) in which the red aggregates (indicated by the arrows) are calcium deposits. The small squares show the photographs taken of each well. Cells treated with osteogenic medium (OS) showed a fully mineralized matrix which means that most hESCs were in a late stage of differentiation to osteoblasts. Those cells treated with BGPN1.3 showed a greater degree of matrix mineralization than those treated either with BG45S5 or DMEM/F12. This study was carried out only for the BG45S5 and BGPN1.3 samples.

The capacity of niobium to stimulate osteogenic differentiation of hESCs was tested by the analysis of matrix mineralization, carried out after 21 days of treatment. This assay revealed the formation of large calcified nodules on the cells treated with BGPN1.3, which suggests cells differentiated into osteoblasts that formed a mineralized bone matrix. These nodules were larger than those observed in the cells treated with BG45S5 **(**Fig. 5**)**.

The products leached from bioactive glass seem to stimulate stem cells to proliferate and differentiate to osteoblasts by activating different intracellular pathways^4,6,44^. Lopes *et al*. observed that the incorporation of up to 1.3% of Nb_2_O_5_ by replacing the P_2_O_5_ in a SiO_2_-Na_2_O-CaO-P_2_O_5_-Nb_2_O_5_ system imposed significant structural changes on the glass, altering its solubility and the rate and magnitude of the Na, Ca, Si and P species leaching from the glass network, compared to niobium-free bioglass^29^.

Although a receptor for Si has not been identified yet, this ion has been shown to be one of the main components of bioglass that is responsible for osteogenic stimulation^4,45,46^. As a component of silicic acid [Si(OH)_4_], silicon has been shown to stimulate osteoblasts to produce collagen I, which is the main component of the extracellular bone matrix^44^. On the other hand, the receptors and intracellular pathways activated by Ca^+2^ in osteoblasts as well as its indirect roles in the bone formation process are very well described^44,47^.

It is well known that calcium possesses both structural and signalling roles in regulating bone homeostasis^44,48–50^. Structurally, hypercalcemia is required to produce mineralized bone as it is combined with PO_4_^−3^, precipitating as calcium phosphate, which constitutes the inorganic phase of the bone matrix. Furthermore, Ca has an important role in regulating bone homeostasis by signalling pathways in bone-forming osteoblasts^44,48,50,51^. It has been reported that this ionic signalling enhances proliferation of stem and mature bone cells through binding to a G-protein, coupled to an extracellular calcium sensing receptor, in a process dependent on nitric oxide (NO) production. Downstream in this signalling, the P13K/Akt pathway is activated, ensuring the surveillance of the osteoblasts^44^.

Niobium species seem to have a direct effect on cells. This fact was demonstrated by Obata *et al*. by treating mouse osteoblast-like cells (MC3T3-E1 cell) with media containing different concentrations of niobium ions (between 1 $\times$ 10^−8^ to 1 $\times$ 10^−5^ M)^25^. Their results showed that, independent of the concentration, niobium did not compromise cell proliferation. Besides this observation, they noted that the ions stimulated a significantly higher alkaline phosphatase activity (APA) in a dose-dependent manner, indicating cell differentiation after 14 days, even in a medium devoid of an osteogenic supplement. When osteogenic media was conditioned with the same ion concentrations, a significant dose-dependent increase was observed in calcium deposition compared to that in the osteogenic medium without niobium ions, suggesting that the niobium ions stimulated greater calcium deposition, which is an indicator of cell maturation. Although we have not studied specific intracellular pathways, we suggest that the greater osteogenic differentiation stimulated by Nb-substituted bioglass observed in our study may have been caused by the presence of niobium species, since the amount of the other bioactive ionic species (based on Si, Ca, Na, P) were similar between the groups. This suggestion corroborates with the results of the aforementioned study^25^.

It is worth mentioning that ICP-OES analysis allowed quantification of the ionized Na, Ca, Si, P, and Nb species released from the glass, but the results do not provide any information on their bioavailability or molecular structure. The dissolution products generated are likely to be in the form of compounds of the elemental constituents of the glass rather than individual ions. Thus, drawing conclusions based only on ion concentration may seem an oversimplification. Obviously, in more complex media, such as culture media or corporeal fluid, the presence of chelating molecules and the ionic strength of the medium may alter the release profile and bioavailability of these species. In order to determine the levels of each element and its bioavailability, the quantification of the leached species from the vitreous matrix, concomitant with molecular speciation studies in a complex, more realistic, media, would be an ideal study, but is a technically challenging experiment. Further studies should focus on determining specifically which compounds are released, which intracellular pathways take part in the osteogenic process, and which genes are regulated by the presence of the bioglass-derived compounds.

### Bone formation stimulated by Nb-containing bioactive glass

*In vivo* experiments were performed in order to analyse the bioactive properties of osteostimulation and osteoconductivity of different Nb-containing glass compositions. Glass rods were implanted into the tibia in rats to test their biological properties by quantifying the newly formed subperiosteal bone and the thickness of the bone layer formed over and near the surface of the rod.

**Figure 6:**
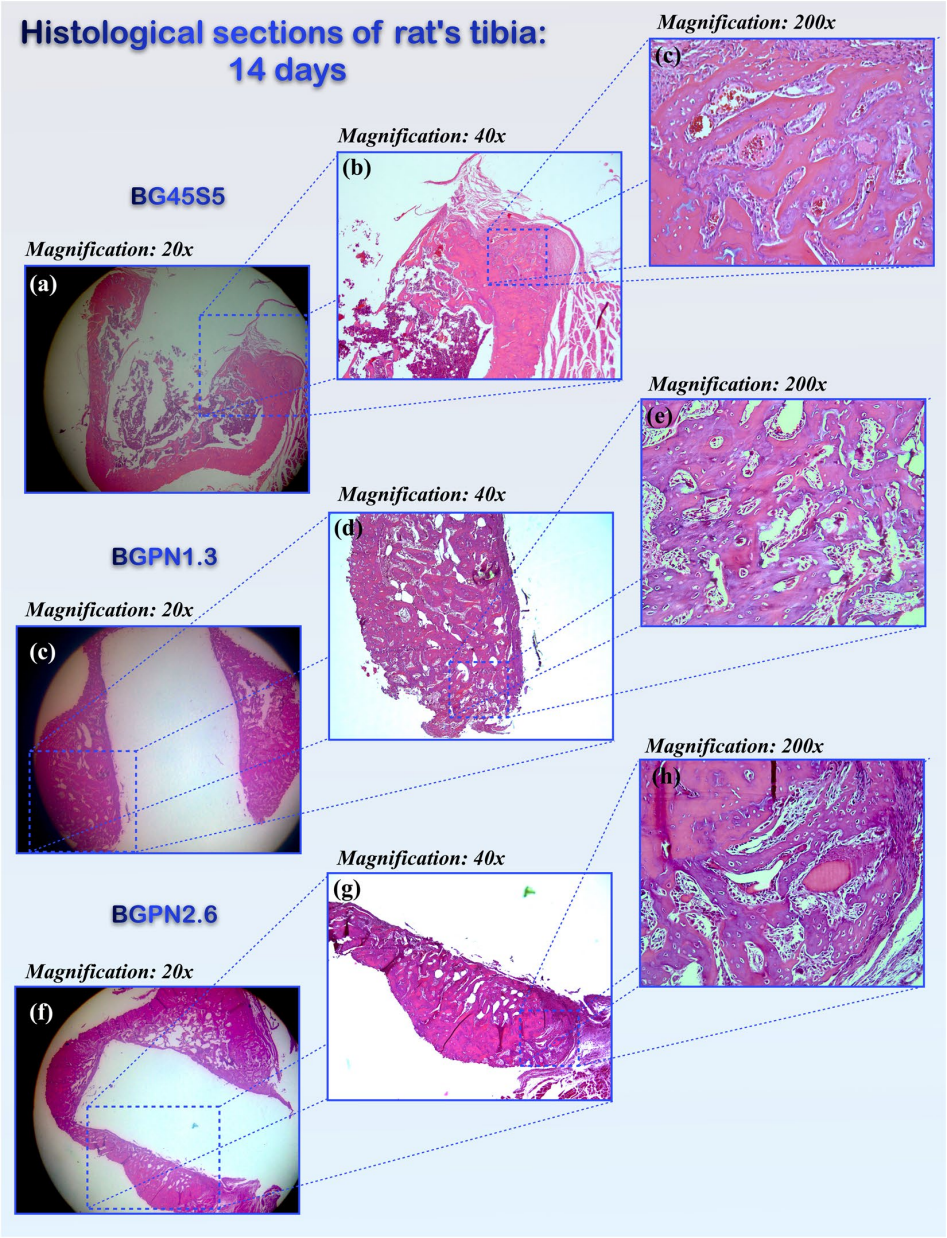
Micrographs of transversal histological sections of rat tibia tissues stained with Haematoxylin & Eosin after 14 days of implantation with different glass compositions. All groups showed similar amount of subperiosteal bone formation [F(3,16) = 1,881, p = 0,174, ω = 0,34].

**Figure 7:**
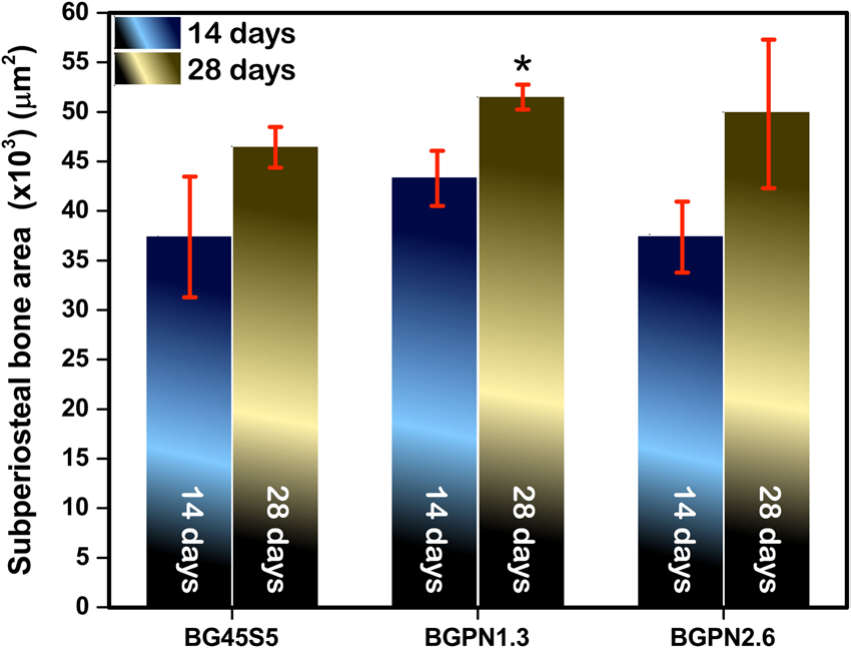
Quantitative analysis of the area of newly-formed subperiosteal bone in rats treated with the different bioactive glass. After 28 days the group treated with BGPN1.3 showed significantly greater bone formation than the control group (treated with BG45S5). All data are displayed as mean and SEM. For all comparisons p < 0.005 was considered.

Comparison of subperiosteal bone area in the rod group tibia and those in a control group showed no significant difference after 14 days of implantation [F (3, 16) = 1.881, p = 0.174)] (Figs 6 and 7).

**Figure 8:**
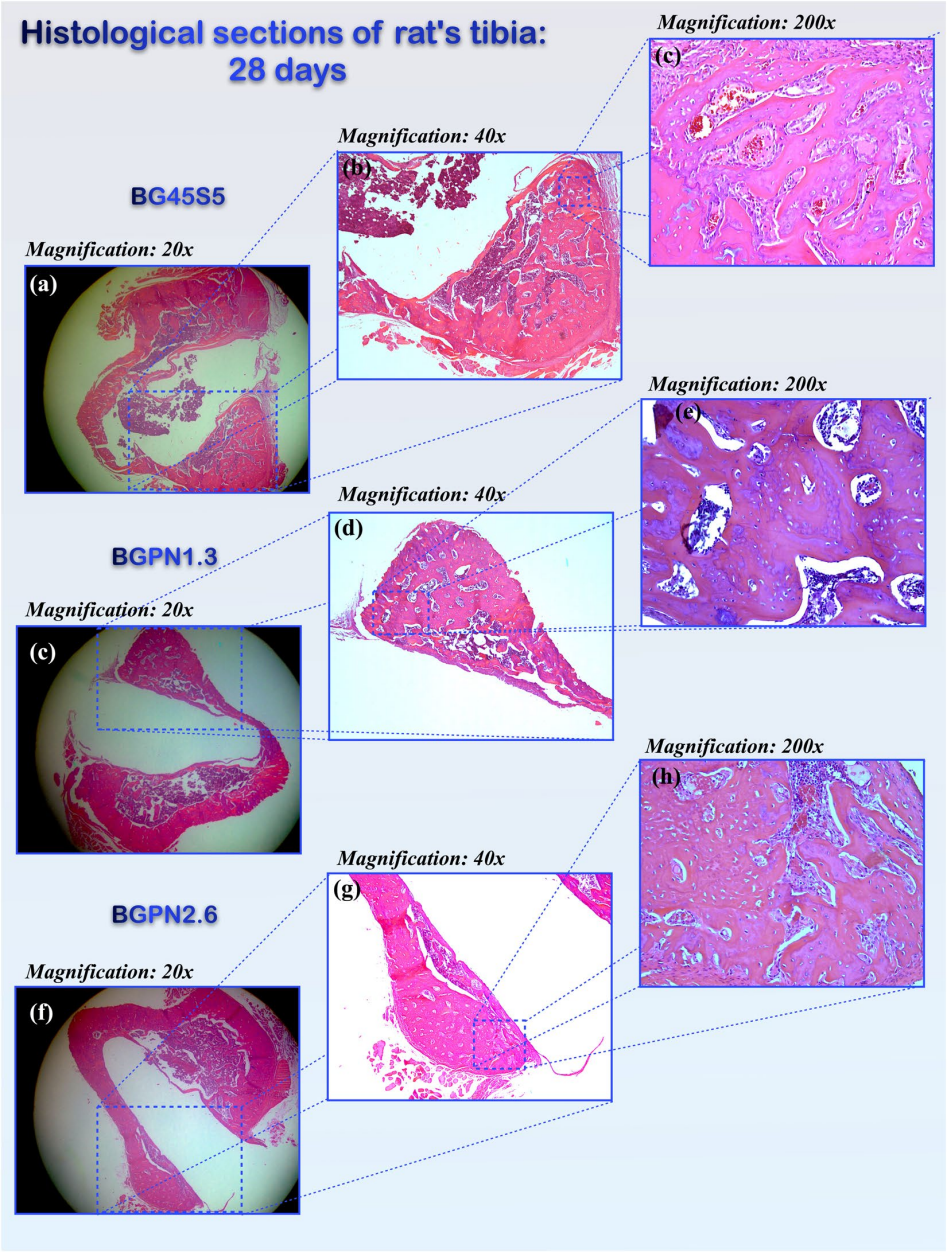
Micrographs of transversal histological sections of rat tibia tissues stained with Haematoxylin & Eosin after 28 days of implantation of different glass compositions. The groups differed regarding newly-formed bone area [F(3,16) = 6,375,p = 0,005, ω = 0,67]. The group BGPN1.3 showed greater bone area (51513,88 ± 1265,13 μ2) than BG45S5 (46537,19 ± 2050,08 μ2) (p = 0.011) and BGSN1(40614,49 ± 3360,57 μ2) (p = 0.004) as can be seen on the micrographs in the right column (200x magnification).

However, after 28 days, BGPN1.3 induced a significant effect on bone formation [F (3, 16) = 6.375, p = 0.005]. The tibia bones in which BGPN1.3 was implanted showed greater subperiostal bone area (51513.88 ± 2050.08 µ^2^) than the control (BG45S5) (46537.19 ± 2050.08 µ^2^) (Figs 7 and 8).

**Figure 9:**
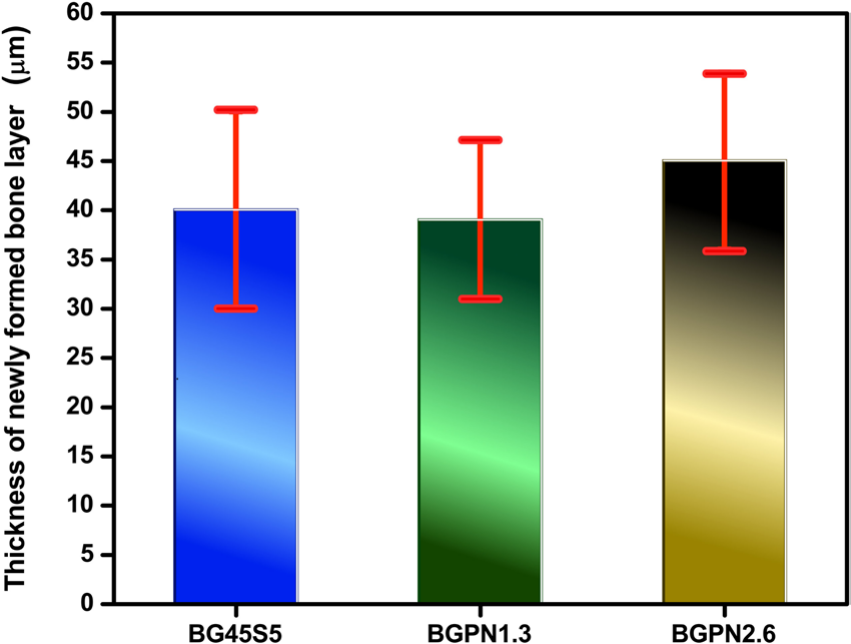
Average thickness of newly-formed bone layer on the surface of different compositions of glass implants after 28 days post-surgery. All values are expressed in mean and SEM and represent thickness in micrometres (µm). There was no significant difference between groups [F(3,16) = 0.423, p = 0.739, ω = 0.31] showing they were both osteoconductive.

Moreover, the presence of niobium did not alter the osteoconductivity of the glass, stimulating the formation of a bone layer over its surface, displaying thicknesses similar to the control group [F (3,16) = 0.423, p = 0.739] (Fig. 9).

We believe the greater bone formation stimulated by Nb-containing glass was caused by the release of Nb species that could have acted in two ways: (a) directly acting over cells by the mechanism explained by the mechanism explained previously in the *in vitro* section of this work and (b) by acting as nucleation agent, forming microscopic needle-shaped crystals on the surface of the glass^52^. This confers a microscopic complexity to the glass surface, which is thought to be critical for *in vivo* contact osteogenesis to occur^24^. It is stated that a micro or nanoscopic complexity allows greater protein, mainly fibrin, adsorption, and that platelets aggregate on this fibrin net and secrete cytokines that recruit osteogenic cells to the implant site^53^. These cells differentiate in osteoblasts and produce a collagen-rich matrix which is further calcified, and newly formed bone is strongly anchored to the surface of the material with an interfacial bond strength equal to or greater than the natural bone^7^. This mechanism explains the osteoconductivity displayed by all glass evaluated in the present study.

We believe that the greater subperiosteal bone formation of the different Nb glass formulations was caused by a direct effect of the leached ionic products (Si, Ca, Na, and P) on the periosteal osteogenic cells, possibly throughout the mechanisms previously discussed in the section “Osteogenic potential of Nb-containing bioglass” of this paper. In short, the leached ionic products may ultimately have caused the upregulation of the expression of important genes for cellular differentiation and maturation. For example, extracellular calcium ions are known to increase the upregulation of IGF-II, one of the main growth factors for bone formation^50,51^.

## Conclusion

To summarise, our results showed that the addition of Nb_2_O_5_, replacing P_2_O_5_, within glass networks increased bioactivity of the glass. All glass compositions derived from Bioglass^®^ tested in the present study exhibited similar release profile for Si, Na and Ca species compared to BG45S5, in addition to the release of niobium species by the compositions of Nb-containing glass. The release of niobium species may have been responsible for the observed enhanced osteogenic and osteostimulative properties.

Solid state ^31^P MAS NMR proved to be a very sensitive technique to elucidate the *in vitro* chemical reactivity and bioactivity of niobium bioactive glass. ^31^P MAS NMR showed that glass containing lower quantities of Nb_2_O_5_ (BGPN1.3) exhibited enhanced early glass apatite formation, whereas for glass compositions containing higher amount of Nb_2_O_5_ (BGPN2.6) the opposite effect was observed.

Nb-substituted silicate glass is not toxic to hESCs and does not cause any problems for liver and kidneys of rats, proving their biocompatibility. The results suggested that hESCs differentiated toward osteoblast lineage after treatment with the dissolution products of Nb-containing glass, revealing the osteogenic capacity of niobium. *In vivo* experiments showed that after 28 days, Nb-containing bioglass induced a significant effect on bone formation.

Based upon these results, we suggest Nb-substituted silicate glass may be an interesting alternative for biomedical applications.

## Materials and Methods

### Preparation of bioglass samples

Details on the preparation of bioglass samples can be found in our previous publications^9,29,31,32^. High purity SiO_2_, Na_2_CO_3_, CaCO_3_, P_2_O_5_ powders (>99.9%), were weighed to obtain the glass compositions depicted in Table 1. All precursor reagents were purchased from Sigma-Aldrich (St. Louis, MO, USA), except for niobium oxide (Nb_2_O_5_, optical grade, >99.5%), which was donated by the CBMM (*Companhia Brasileira de Metaluria e Mineração*, *Araxá*, *Minas Gerais*, *Brazil*). Thirty-gram batches were melted in a platinum crucible at 1400 °C in air for 3 hours using a furnace (Lindberg/Blue M 1700 °C, Thermo Electron Corporation, Asheville, NC, USA). At the end of the refining process, the melts were quenched directly into cold water (frit cast), collected in a sieve, and dried to constant weight at 120 °C overnight. The different types of glass were then ground using an agate mortar and pestle, and sieved to separate fine (<38 µm) and coarse (>53 µm) particles. All experiments described in this work were carried out using glass particles size between 38–53 µm, excepting the *in vivo* studies for which cylindrical rods with 4 mm length × 2 mm diameter were used. These samples were prepared by casting method in a graphite mould (Fig. SI2). Glass rods were annealed at 50 °C below the glass transition temperature (*T*_*g*_) for 12 h and slowly cooled to room temperature to release the thermal stress associated with the quenching process.

### Ion leaching experiments by ICP-OES

The ionic leaching patterns of the bioactive glass were determined by inductively coupled plasma optical emission spectrometry (ICP-OES). In this experiment, 300 mg of bioglass particles were dispersed in a beaker with 200 mL of buffer solution and maintained under continuous stirring (stirring rate of 75 rpm), at room temperature. The solution was buffered at pH 7.40 by using 50.69 mM HEPES (2-(4-(2-hydroxyethyl)piperazin-1-yl)ethanesulfonic acid) and 1 mM NaOH, both purchased from Sigma-Aldrich (St. Louis, MO, USA). Prior to the addition of the glass particles, 50.69 mM HEPES solution was passed through a 0.22 μm Millipore^®^ filter to remove eventual aggregates or contaminants. During the leaching experiment, the beaker was kept sealed with parafilm in order to avoid any loss of liquid by evaporation, preventing changes in the ratio of glass/HEPES in the solution. After 3, 6, 12, 24 and 48 hours, 10 mL of solution was removed with a syringe. Since the powder suspension was well dispersed and homogenous, the sample removal did not alter the solution concentration. For this reason, the solution was not refilled after collecting samples, resulting in a final volume of the solution of 150 mL. After each removal, the solution was immediately filtered with 0, 22 µm Millipore^®^ filters and analysed with an ICP-OES spectrometer (Optima 8300 ICP-OES, PerkinElmer, Inc., Shelton, CT, USA). The calibration curves were obtained from standard solutions containing Ca, Na, P, Si, and Nb. In order to ensure the accuracy of the calibration curve, the standard sample concentrations were measured periodically. Three replicates were measured for each element. The results obtained for the same element from different emission lines were averaged. Each point of the graphs shown is the result of the average of three dissolution experiments. Elemental concentration was reported in ppm.

### Formation rate of calcium phosphate layer determined by ^31^P MAS NMR Spectroscopy

The rate of formation of the calcium phosphate layer on bioglass surfaces was monitored by ^31^P MAS NMR. Samples were prepared by adding 50 mg of bioactive glass particles to 5 mL of simulated body fluid (SBF)^54^ (see supplementary information – Table SI1), and stirring in a thermostatic bath at 37 °C. After 3, 12, 24 and 48 hours the solution was filtered and the remaining powder was immediately dried between two filter papers. Soon after the drying procedure the material was packaged in the rotor and analysed by ^31^P MAS NMR.

^31^P MAS NMR study was carried out using an FT-NMR (AVANCE II + 400, 9.04 T, Bruker, Rheinstetten/Karlsruhe, Germany) at the resonance frequency 161.98 MHz, using standard Bruker double-resonance magic-angle sample spinning (MAS) probes. The glass particles were packed into a 4-mm cylindrical zirconia rotor and spun at the MAS frequency at the magic angle to remove any anisotropy effects. The samples were spun at 10 kHz at the ambient probe temperature. The ^31^P MAS spectra were obtained using a high-power decoupling (HPDEC) pulse sequence with 2.50 μs pulses, 82 ms acquisitions, 5 s recycle delays, a 100 kHz spectral width, and 1024 scans. The ^31^P chemical shifts were referenced to 85% H_3_PO_4_. The spectra were processed with TopSpin 2.1.6 software using Fourier transforms and an exponential filter of 50 Hz. The phase was manually adjusted, and the baseline was obtained using a five-order polynomial function.

### Culture of human embryonic stem cells (hESCs)

Human Embryonic Stem Cells (hES cells) CCTL12 were cultured in complete human embryonic stem cell medium (cHES) composed of Dulbecco’s Modified Eagle Medium Nutrient Mixture F-12 (DMEM/F-12) supplemented with 2 mM of L-glutamine (Gibco^®^, Thermo Fisher Scientific Inc.) and 10 ng/mL of fibroblast growth factor 2 (FGF2, PepTech Corporation, Bedford, MA, USA). Before being used with hES cells, cHES was conditioned by incubation for 24 h with confluent cultures of mouse embryonic fibroblasts.

For all cell experiments the hES cells were cultured in flasks previously coated with BD Matrigel™ and using one of the following four culture media: (a) cHES; (b) Osteogenic Medium (OS), composed by cHES supplemented with 3 mM β-glycerolphosphate, 0.1 mM ascorbic acid and 10^−8^ M dexamethasone (Sigma-Aldrich, St. Louis, MO, USA); (c) BG45S5 medium, in which BG45S5 powder was mixed with cHES medium at 10 mg/mL; and (d) BGPN1.3 medium, in which Nb-substituted bioglass was mixed with cHES at a concentration of 10 mg/mL. All glass-conditioned media were sterilized using a 0.22 µm pore filter (which also removed the powder particles) prior treating the cells. Cells were incubated at 37 °C in a humid 5% CO_2_ atmosphere.

### Cell viability – resazurin assay

Cell viability was determined by means of resazurin assay (Sigma-Aldrich, St. Louis, MO, USA). The culture medium was supplemented with 5 µM of resazurin and the cells were incubated at 37 °C and 5% CO_2_ for 4–6 hours. The total metabolic activity was measured by scanning the cell plate at a plate reader (Synergy HT, BioTek^®^ Instruments, Inc, Winooski, VT, USA) on the fluorometry mode with excitation filters centred at wavelengths of 540 nm and 590 nm.

### Matrix mineralization

The Alizarin red stains intracellular calcium deposits and calcium bound to proteins and proteoglycans^55,56^. This dye was used to analyse the deposition of mineralized matrix by osteoblasts, which is a marker of cell maturation. The test was conducted after 21 days of cell culture (the time required for observing matrix mineralization) within the four different experimental media. For the test, the cell monolayers were fixed with 70% ethanol for 1 hour at 4 °C and incubated in 1% Alizarin Red (Sigma-Aldrich, St. Louis, MO, USA) for 10 minutes. After this period five washes in distilled water were carried out and microphotographs at 100x magnification were taken from each well. Each treatment was applied to five wells per plate and the whole assay was performed in duplicate.

### *In Vivo* assessment

In order to test the biological response incited by the bioactive glass, glass rods were implanted into rat tibia. For this, Brazilian College of Animal Experimentation (BCAE) guidelines were followed, and experiments were approved by Committee for Ethics in Animal Use of the University of Campinas - CEUA/Unicamp (Protocol n° 2777–1). Rods composed of different types of glass (BGPN1.3, BGPN2.6, and BG45S5) were implanted into a round defect created in the tibia of rats that had been anesthetized with a mixture of ketamine (ANASEDAN^®^) at 80 mg/kg and xylazine (DOPALEN^®^) at 10 mg/kg. Five rats were used per group. After pulling aside the periosteum a round transcortical defect was created using a spherical threfine bur (JET®) with a diameter of 2 mm. The glass rod (4 mm length × 2 mm diameter) was carefully introduced into the defect until total coupling, then the periosteum and the skin were sutured. After 14 or 28 days, the animals were euthanized, and the tibia was obtained from the animals and fixed for 24 h at 4 °C in 10% zinc-buffered formalin (Sigma-Aldrich®). After fixation, they were decalcified in 5% EDTA (Synth Labsynth, Diadema, SP, Brazil), the glass rod was then carefully removed and the bones were paraffin-embedded and sectioned.

### Systemic toxicity

To assess the systemic toxicity of the glass implants we calculated the relative weights of rats’ liver and kidneys. For this, immediately after euthanasia (14 or 28 days after implantation), liver and kidneys were dissected from the five rats of each group and weighed with a high precision scale (Unibloc®). The organ relative weights were calculated as the ratio between organ weight and body weight.

### Morphometry

All histological sections were stained with haematoxylin and eosin dyes. A light microscope (80i) with a camera (DS-Ri1) and software (NIS-Elements software Advanced Research 3.0), all made by Nikon Corporation, Tokyo, Japan, were used to quantify the following parameters:

i) **Area of newly-formed subperiostal bone**. The area of the newly-formed subperiostal bone was measured at one field of each side of the cortical defect, directly underneath the periosteum and adjacent to where the glass rod was previously located, at 400x magnification. Five non-consecutive histological sections were analysed per animal. Five rats were used per group (n = 5 per group). Thus, the mean and standard error of the mean were registered and further compared (Fig. SI3).
ii) **Thickness of newly-formed bone layer**. Along the bone layer that formed around the implant we performed 20 measurements of thickness. All measurements were performed at 400x magnification. Five rats were used per group (n = 5 per group). The mean and standard error of the mean were registered and further compared (Fig. SI4).

### Statistical analysis

We used One-way ANOVA followed by Student Newman-Keuls test to compare the organ relative weights. Comparisons between means of subperiostal newly-formed bone and thickness of the bone layer formed around the implant were made using ANOVA one-way test with Tukey (homogeneous variances) or Games-Howell (non-homogeneous variances) post-hoc. For comparisons between the amounts of trabecular bone formed at 14 post-operative days, a Kruskal-Wallis test was used due to the non-normality of the data, matching the control group with each of the other groups through Mann-Whitney tests post hoc. For all tests α = 0.05 was assumed. All data are presented as means and standard errors.

## Electronic supplementary material

Supplementary Information
