# Supplementary material for: Comprehensive in vitro and in vivo studies of novel melt-derived Nb-substituted 45S5 bioglass reveal its enhanced bioactive properties for bone healing
Source: Sci Rep. 2018 Aug 24;8:12808. doi: 10.1038/s41598-018-31114-0 (PMC6109119; doi:10.1038/s41598-018-31114-0)
Supplement: Supplementary file 1 — Supplementary Information [file 41598_2018_31114_MOESM1_ESM.pdf]

# Supplementary Information - SI

# Comprehensive *in vitro* and *in vivo* studies of novel melt-derived Nb-substituted 45S5 bioglass reveal its enhanced bioactive properties for bone healing

*Lucas Souza[1,‡], João Henrique Lopes[2,‡,*], Davi Encarnação[2], Italo Odone Mazali[3], Richard Alan Martin[4] , José Angelo Camilli[1], Celso Aparecido Bertran[2]*

AUTHOR INFORMATION

**L. Souza**

[1]Department of Structural and Functional Biology, Institute of Biology, University of Campinas – UNICAMP, 13083-862, Campinas, SP, Brazil.

E-mail: (lpls2002@hotmail.com)

**J. H. Lopes (Corresponding Author)**

[2]Department of Physical Chemistry, Institute of Chemistry, University of Campinas – UNICAMP, P.O. Box 6154, 13083-970, Campinas, SP, Brazil.

E-mail: (henriquelopez@gmail.com)

**D. Encarnação**

[2]Department of Physical Chemistry, Institute of Chemistry, University of Campinas – UNICAMP, P.O. Box 6154, 13083-970, Campinas, SP, Brazil.

E-mail: (davi.encarnacao1@gmail.com)

**I. O. Mazali**

[3]Department of Inorganic Chemistry, Institute of Chemistry, University of Campinas – UNICAMP, P.O. Box 6154, 13083-970, Campinas, SP, Brazil.

E-mail: (mazali@iqm.unicamp.br)

**R. A. Martin**

[4]School of Engineering & Aston Research Centre for Healthy Ageing, Aston University, B47ET Birmingham, United Kingdom.

E-mail: (r.a.martin@aston.ac.uk)

**J. A. Camilli**

[1]Department of Structural and Functional Biology, Institute of Biology, University of Campinas – UNICAMP, 13083-862, Campinas, SP, Brazil.

E-mail: (jcamilli@unicamp.br)

**C. A. Bertran**

[2]Department of Physical Chemistry, Institute of Chemistry, University of Campinas – UNICAMP, P.O. Box 6154, 13083-970, Campinas, SP, Brazil.

E-mail: (bertran@iqm.unicamp.br)

**Correspondence to henriquelopez@gmail.com*

*‡ These authors contributed equally.*

## *SI1. Ion release in 50.69 mM HEPES solution at pH 7.40 for BG45S5 and Nb-substituted bioactive glass*

**Figure SI1** shows a linear function with the square root of time up to about 120 min for all compositions.

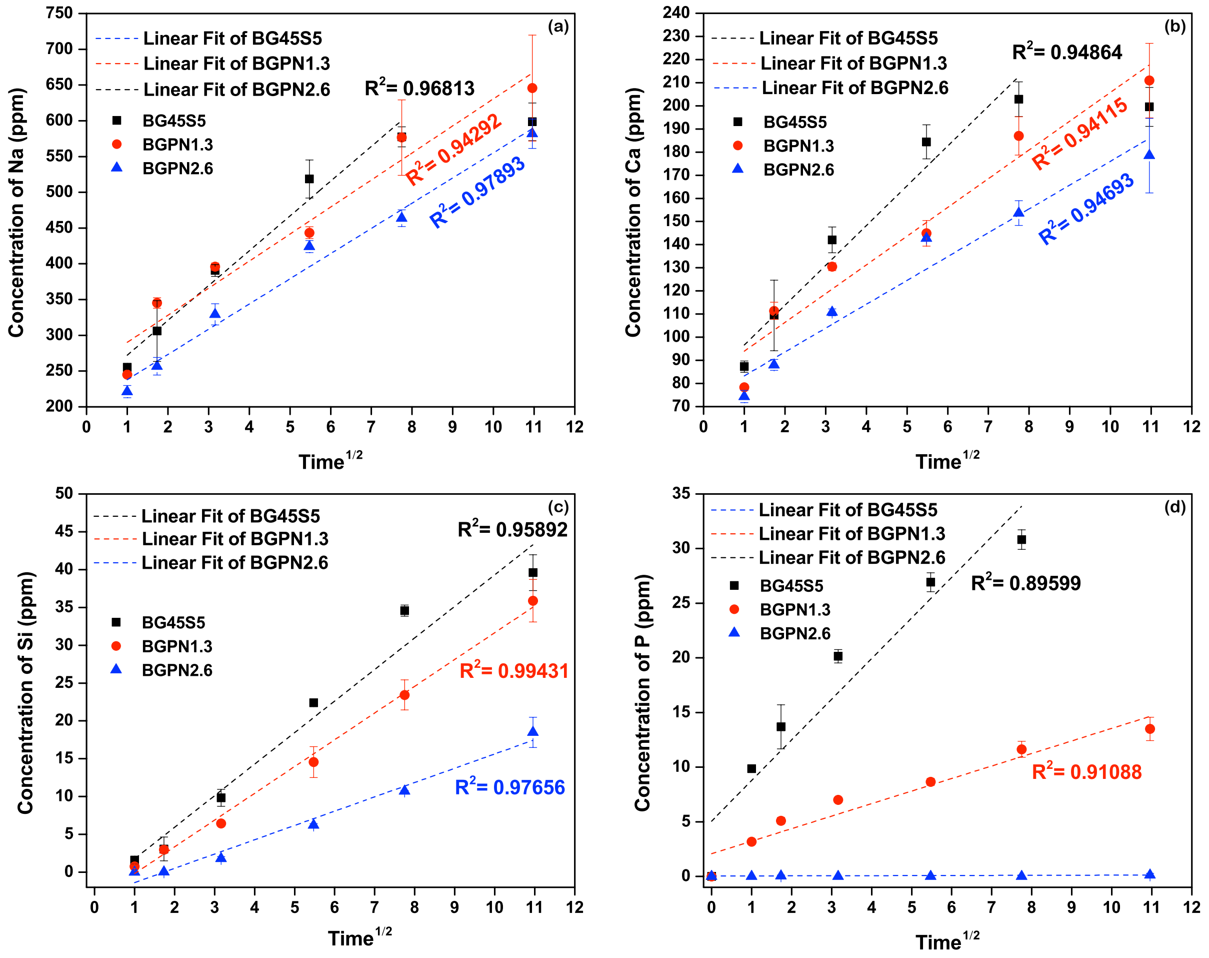


**Figure SI1.** ICP data: ion release as a function of square root of time in 50.69 mM HEPES solution at pH 7.40 for BG45S5 and Nb-substituted bioactive glass. The data displayed in (a), (b), (c), and (d) are related to leached of Na, Ca, Si, and P species, respectively, from glass derived from substitution of $P_2O_5$ by $Nb_2O_5$. Lines are linear regression of dissolution data up to 120 min.

The elemental concentrations of sodium, calcium, silicon, and phosphorus exhibited a linear function of the square root of time up to about 120 min for all compositions, which indicates a two-step degradation mechanism with initial dissolution controlled by diffusion (exhibits a $t^{1/2}$ dependence).

## *SI2. Glass rods preparation*

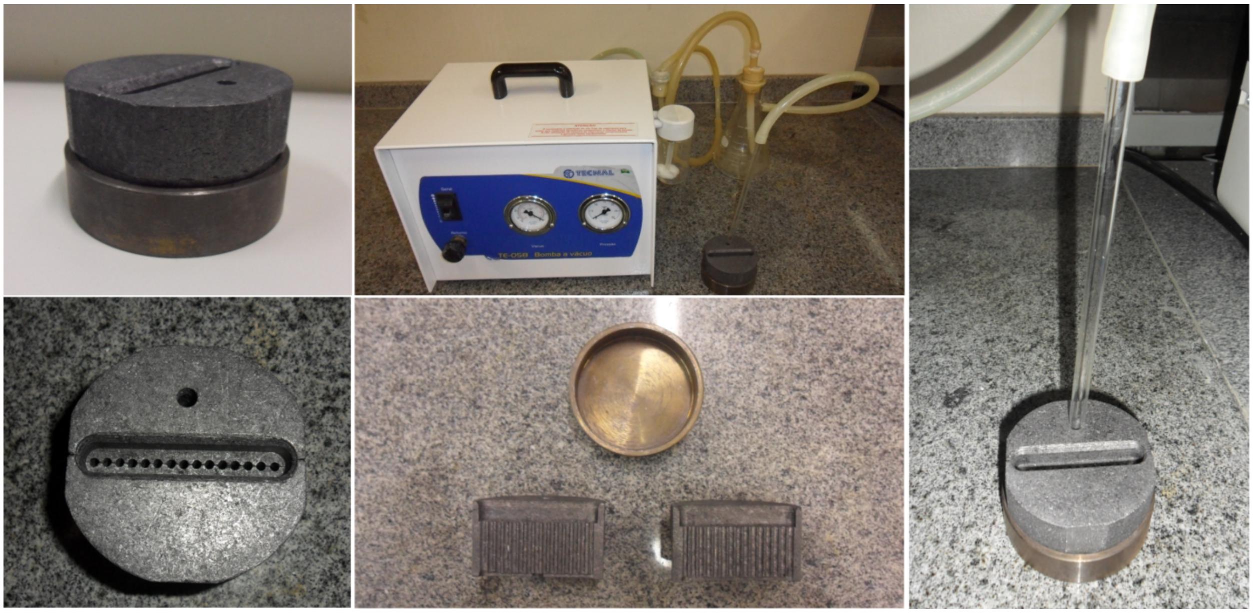

**Figure SI2.** Devices used for the preparation of glass rods for in vivo tests: Graphite mould and suction system to force filling of the glass in the graphite mould. The vacuum pump was used to suction the molten glass, overcoming the high surface tension of the melt and allowing it to fill the 2 mm diameter graphite mould.

## *SI3. Preparation of SBF solution*

The acellular simulated body fluid (SBF) solution was prepared with concentrations of ions equal to those of blood plasma. The pH value of 7.40 was obtained using the Hepes buffer at concentration 50.69 mmol/L. Details about the concentrations of the ions present in the SBF solution is presented in **Table SI1.**

**Table SI1.** Nominal ion concentrations of the SBF in comparison with those of human blood plasma.

| **Ions** | **Concentration/mM** | |
|---|---|---|
| | **Blood Plasma** | **SBF** |
| $Na^+$ | 142.0 | 142.0 |
| $K^+$ | 5.0 | 5.0 |
| $Mg^{2+}$ | 1.5 | 1.5 |
| $Ca^{2+}$ | 2.5 | 2.5 |
| $Cl^-$ | 103.0 | 103.0 |
| $HCO_3^{2-}$ | 27.0 | 27.0 |
| $HPO_4^{2-}$ | 1.0 | 1.0 |
| $SO_4^{2-}$ | 0.5 | 0.5 |

***SI4.*** **Morphometry**

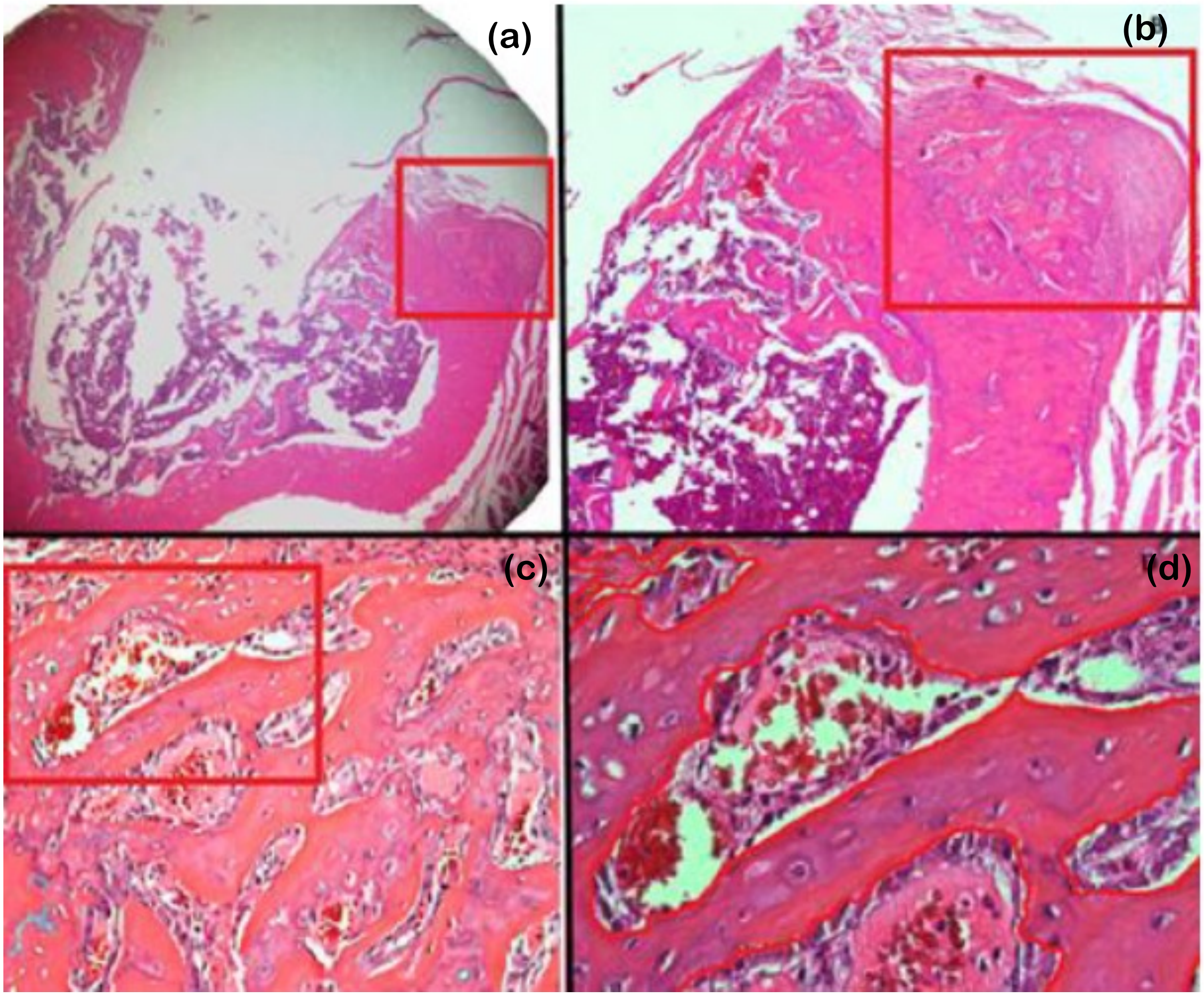


**Figure SI3.** Micrographs showing H&E stained histological sections of rat tibia tissues at 40×, 100×, 200×, and 400× magnifications and quantification of the area of newly-formed subperiostal bone. The area of the newly-formed subperiostal bone was measured in one field of each side of the cortical defect, directly underneath the periosteum and adjacent to where the glass rod was previously located at 400× magnification. Five non-consecutive histological sections were analysed per animal. Five rats were used per group (n = 5 per group). Thus, the mean and the standard error of the mean were recorded and further compared.

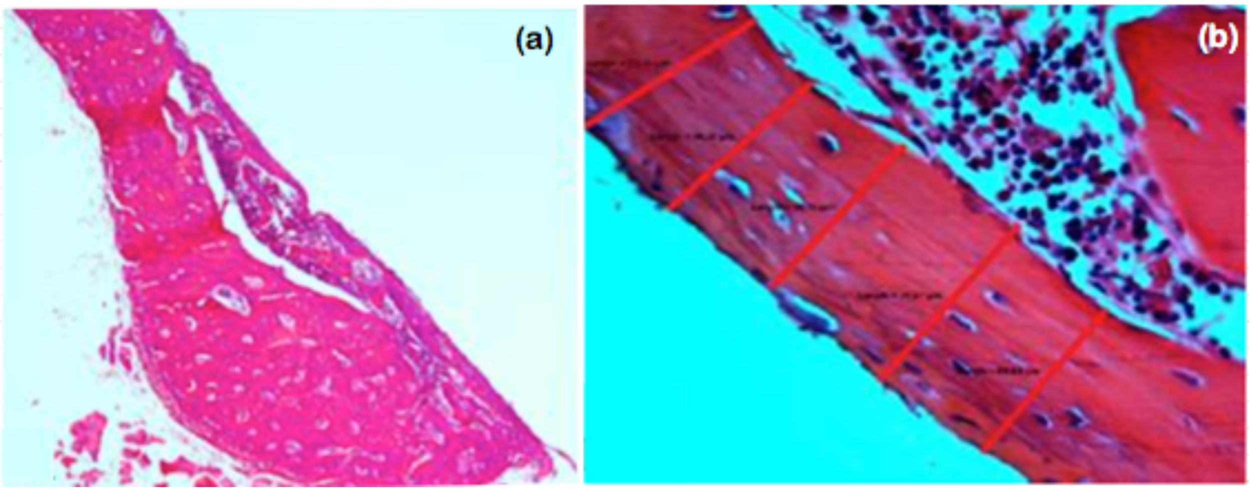


**Figure SI4.** Micrographs showing H&E stained histological sections of rat tibia at 40×, 100×, 200×, and 400× magnifications. Measurement of the thickness of newly-formed bone layer that formed around the implant was repeated 20 times, and all measurements were performed at 400× magnification. Five rats were used per group (n = 5 per group). The mean and the standard error of the mean were recorded and further compared.
